# Long-term impact of childhood malaria infection on school performance among school children in a malaria endemic area along the Thai–Myanmar border

**DOI:** 10.1186/s12936-015-0917-7

**Published:** 2015-10-09

**Authors:** Nutchavadee Vorasan, Wirichada Pan-Ngum, Podjanee Jittamala, Wanchai Maneeboonyang, Prasert Rukmanee, Saranath Lawpoolsri

**Affiliations:** Department of Tropical Hygiene, Faculty of Tropical Medicine, Mahidol University, Bangkok, Thailand

**Keywords:** Malaria, Cognitive, School performance, Nutritional status, Emotional intelligence

## Abstract

**Background:**

Children represent a high-risk group for malaria worldwide. Among people in Thailand who have malaria during childhood, some may have multiple malaria attacks during their lifetime. Malaria may affect neurological cognition in children, resulting in short-term impairment of memory and language functions. However, little is known regarding the long-term effects of malaria infection on cognitive function. This study examines the long-term impact of malaria infection on school performance among school children living in a malaria-endemic area along the Thai–Myanmar border.

**Methods:**

A retrospective cohort study was conducted among school children aged 6–17 years in a primary-secondary school of a sub-district of Ratchaburi Province, Thailand. History of childhood malaria infection was obtained from the medical records of the sole malaria clinic in the area. School performance was assessed by using scores for the subjects Thai Language and Mathematics in 2014. Other variables, such as demographic characteristics, perinatal history, nutritional status, and emotional intelligence, were also documented.

**Results:**

A total of 457 students were included, 135 (30 %) of whom had a history of uncomplicated malaria infection. About half of the malaria-infected children had suffered infection before the age of four years. The mean scores for both Mathematics and Thai Language decreased in relation to the increasing number of malaria attacks. Most students had their last malaria episode more than two years previously. The mean scores were not associated with duration since the last malaria attack. The association between malaria infection and school performance was not significant after adjusting for potential confounders, including gender, school absenteeism over a semester term, and emotional intelligence.

**Conclusions:**

This study characterizes the long-term consequences of uncomplicated malaria disease during childhood. School performance was not associated with a history of malaria infection, considering that most students had their last malaria infection more than two years previously. These findings indicate that the impact of uncomplicated malaria infection on school performance may not be prolonged.

## Background

Malaria infection remains a major public health problem worldwide. The World Health Organization has estimated that malaria affects 198 million individuals, and globally 584,000 persons died from the disease in 2013 [1, 2]. Children are at high risk of malaria infection and are more likely to have serious conditions once infected [1–4]. In addition, malaria may affect neurological and physical development in children [5–9], especially when infection occurs in young children [10–13]. Malaria infection may affect cognitive performance, which leads to impairment of memory and language functions [10–13]. Previous studies suggested that childhood malaria infection is associated with poor school performance [14–18]. Malaria infection is an important cause of school absenteeism among African children, which may affect their school performance [3]. In African children, impaired cognitive function can persist up to two years after cerebral malaria episode [3]. Moreover, multiple attacks of uncomplicated malaria in childhood may also affect cognitive function and school performance [12]. However, most findings were based on cross-sectional or cohort studies of short duration (1–2 years), which only represent the short-term impact of malaria infection on cognitive functions in children [14–18]. In addition, data on long-term cognitive impairment after malaria infection are derived mainly from studies of children with severe malaria [8, 9]. There is still a lack of evidence regarding the long-term impact of uncomplicated malaria infection on cognitive function among children in settings of low endemicity where a relatively large at-risk population resides [19].

Thailand is considered as a malaria-endemic country. Although the overall endemicity is low nationwide, the transmission is most intense in rural areas along the international borders. In these areas malaria remains the major cause of illness among children, many of whom may experience multiple malaria attacks during their childhood [20]. However, there is no available evidence regarding the impact of childhood malaria infection on the cognitive function of these children, especially over the long term. Therefore, this study aimed to determine the impact of malaria by means of a retrospective cohort study on school performance over more than 10 years among school children in a primary-secondary school located in an area of low malaria endemicity along the Thai–Myanmar border. The longitudinal data of the student cohort allow assessment of the long-term impact of malaria infection on cognitive function through analysis of school performance. Findings from this study should provide further insight into the consequences of childhood malaria infection among children living in areas of low malaria endemicity.

## Methods

### Study area

The study was conducted in Tanousri, Suanphuang, Ratchaburi Province, located on the western border of Thailand. This is a mountainous area bordering Myanmar, where malaria transmission persists throughout the year. The Rajanagarindra Tropical Diseases Center (RTIC) operated by Mahidol University, the sole malaria clinic in this area, has provided free diagnosis and treatment of malaria to people in this district for more than 15 years. The vast majority of malaria cases in the area are detected at this center. There is only one primary-secondary school in Tanousri, which provides free tuition and education to approximately 1000 students.

### Study population

Children aged 6–17 years old who were studying in 2014 at the Rujirapat primary-secondary school of Tanousri, Suanphuang, Ratchaburi Province, Thailand, and were permanent residents in the area, were enrolled in the study. Students who had a history of meningitis, encephalitis, and other central nervous system infections were excluded.

### Study design

A retrospective cohort study was conducted among students in the school. History of malaria infection since birth was obtained from the medical records of the RITC. All malaria cases were based on microscopic diagnosis by trained and experienced staff. The participating students were classified into malaria and non-malaria groups according to their history of malaria infection. History of intestinal parasitic infection was obtained from the most recent mass stool examination survey in 2013 performed by trained personnel. Information on school performance (outcome) and other variables such as demographic characteristics, perinatal and birth history, nutritional status, and emotional intelligence were primarily collected in 2014.

### Assessment of school performance

School performance was assessed using pupils’ scores in the subjects Mathematics and Thai Language, graded by class teachers and ranging from 0 to 100. The education tests were routinely developed following a standard protocol of the school, which regularly received quality assurance by the Ministry of Education, Thailand. Students in the same grade were tested with the same set of paper examination. The level of school performance was classified according to a standardization protocol: poor school performance was identified when a z-score test was below zero [17]. This standardization technique helps to avoid bias that might occur due to variation in standards of grading by different teachers; different teachers may have different evaluation standards that may lead to variation in test scores of children in different classrooms.

### Assessment of nutritional status

Weight and height of students were collected from school records, documented routinely by the school. The measurement was performed by trained teachers following standard procedures provided by the Ministry of Public Health. Nutritional status was assessed following the standard guidelines of WHO, including weight for age and height for age. Poor nutritional status was considered when weight for age and height for age was below −2.0 of the standardized z-score [21, 22].

### Assessment of emotional intelligence

Emotional intelligence was assessed by a standard emotional intelligence test (EQ test) developed by the Ministry of Public Health of Thailand. This standard questionnaire for EQ screening was tested among children in different parts of Thailand, and shown to have high validity and reliability [23]. Following the instructions for the test, children aged 6–11 years were assessed by trained teachers and children aged 12–17 years were assessed by self-evaluation of students.

The outcome of the EQ test was classified according to the guidelines of the Ministry of Public Health of Thailand. Students were classified as having low emotional intelligence if the test score was below 40 out of 100 for students aged 6–11 years and below 140 out of 208 for those aged 12–17 years [24, 25].

### Collection of demographic, socioeconomic, and perinatal history data

Students and their parents were interviewed using a structured questionnaire. Questions concerned basic demographic characteristics and socioeconomic status. Perinatal and birth histories of students were obtained from the review of the perinatal log book (if available).

### Data analysis

All analyses were performed using SPSS version 18.0 (SPSS Inc., Chicago, IL, USA). Significance was considered at an alpha level of less than 0.05 (p < 0.05). The Student t-test and analysis of variance were used to analyse mean differences between groups. Logistic regression was used to determine crude odds ratio (OR) and 95 % confidence interval (CI) for predictive factors of school performance. Factors that showed significant associations were then included in multivariate analysis to estimate adjusted ORs and 95 % CIs.

### Ethical considerations

The study was approved by the Ethical Review Committee of the Faculty of Tropical Medicine, Mahidol University, Thailand. Informed consent and assent forms were obtained from parents and students who participated in the study.

## Results

### General and socioeconomic characteristics

A total of 457 school children were enrolled. About half of the students were male (52 %) and in the age group 6–11 years (53 %). The majority of students (82 %) were Karen, an ethnic minority that live along the Thai–Myanmar border. Most fathers and mothers of students were uneducated (60 and 70 %, respectively), and 67 % of students were from low-income families (<30,000 baht per year). More than 70 % of students were term delivered with a birth weight of 2500 g or more (Table 1). Although almost all students had no complications at birth, some had a history of breech presentation and neonatal jaundice.

**Table 1:** General and socioeconomic characteristics of schoolchildren

Variable	N	Percentage
Total	457	
Sex		
Male	237	51.9
Female	220	48.1
Age (years)		
6–11	244	53.4
12–17	213	46.6
Ethnicity		
Karen	374	81.8
Thai	83	18.2
Perinatal history		
Birth weight (grams)		
<2500	123	28.4
≥2500	310	71.6
Gestational age		
<36 weeks	120	27.6
≥36 weeks	315	72.4
Father’s education		
Uneducated	272	59.5
Educated	185	40.5
Mother’s education		
Uneducated	323	70.7
Educated	134	29.3
Family income per year (baht)		
<30,000	306	67.0
≥30,000	151	33.0
History of malaria infection		
Non-malaria infected	322	70.5
Malaria infected	135	29.5
Results of Intestinal parasitic infection (only those detected; n = 202)		
No infection	104	51.5
Infection	98	48.5
Nutritional status		
Weight for age		
Normal	406	88.8
Poor nutritional status	51	11.2
Height for Age		
Normal	392	85.8
Poor nutritional status	65	14.2
School absenteeism (during a semester term)		
<1 week	372	81.4
≥1 week	85	18.6
Emotional intelligence		
Good	82	17.9
Moderate	195	42.7
Low	180	39.4

Most students had normal nutritional status in terms of weight for age and height for age. However, more than one-third (39 %) had low emotional intelligence. Most students attended the school regularly; only 19 % accumulated absenteeism of seven days or more. A total of 202 students were tested for intestinal parasitic infection in 2013. Among them, 98 (49 %) had been infected with intestinal parasites and subsequently received treatment (Table 1). The prevalence of parasitic infection was hookworm 24 %, *Ascaris lumbricoides* 8 %, *Giardia lamblia* 7 %, *Trichuris trichiura* 2 %, *Enterobius vermicularis* 2 %, and *Opisthorchis viverrini* 2 % respectively. Approximately 30 % of children had infected with more than one species of parasite.

A history of childhood malaria infection was recorded in 135 students (30 %), all of whom had uncomplicated malaria. The majority of malaria cases were infected with either *Plasmodium falciparum* or *Plasmodium vivax*. Only two students were infected with *Plasmodium malariae.* Among these children, 99 (73 %) had experienced 1–2 malaria attacks and 13 (17 %) had had five or more malaria attacks since birth. Almost half of the malaria-infected students (45 %) had their first malaria infection during the first four years of life. Recent infection (within <1 year) was observed in only 4 students. For the majority of malaria-infected students (80 %) it had been three years or more since their last malaria infection (Table 2).

**Table 2:** Pattern of malaria infection among malaria-infected students

Variable	N	Percentage
Total	135	
Type of malaria infection		
*Plasmodium falciparum* (PF)	50	37.0
*Plasmodium vivax* (PV)	45	33.4
*Plasmodium malariae* (PM)	1	0.7
Mixed infection (PF and PV)	5	3.7
PF + PV*	33	24.5
PM + PV*	1	0.7
Number of malaria attacks		
1–2	99	73.3
3–4	23	17.0
≥5	13	9.6
Age at first malaria infection (years)		
0–4	61	45.2
>4	74	54.8
Duration since last malaria attack (years)		
<1	4	3.0
1–2	19	14.1
3–4	33	24.4
≥5	79	58.5

* Multiple episodes

### Association between childhood malaria infection and school performance

Mean scores for both Mathematics and Thai Language significantly decreased in relation to the increasing number of malaria attacks (Table 3). Students who had a history of five or more malaria episodes had lower scores in Mathematics and Thai Language than those who had only one or two malaria episodes. However, the mean score in Mathematics and Thai Language showed no statistically significant difference regarding the length of time since the last malaria infection (Table 4).

**Table 3:** Mean and standard deviation of school performance by number of malaria attacks

Variable	Number of malaria attacks				p
	0 (n = 322)	1–2 (n = 99)	3–4 (n = 23)	≥5 (n = 13)	
School performance					
Mathematics score	72.1 (13.7)	70.0 (12.9)	65.5 (20.6)	57.6 (26.7)	0.001
Thai language score	74.5 (10.9)	73.7 (8.37)	73.0 (8.32)	69.2 (7.37)	0.04

**Table 4:** Mean and standard deviation of school performance by duration since the last malaria infection

Variable	Duration since last malaria infection (years)				p
	<1 (n = 4)	1–2 (n = 19)	3–4 (n = 33)	≥5 (n = 79)	
School performance					
Mathematics score	76.5 (8.8)	69.6 (13.8)	67.3 (15.7)	67.7 (17.2)	0.72
Thai language score	73.5 (10.1)	72.2 (6.8)	72.5 (9.45)	73.8 (8.26)	0.84

### Factors associated with school performance

School performance was identified according to the standardized score. Of 457 students, 211, 220, and 168 students had poor scores in Mathematics, Thai Language, and both, respectively. Factors associated with poor school performance were firstly identified separately for each Mathematics or Thai Language test. The results of each individual analysis showed similar patterns regarding the predictive factors. Therefore, only the results of school performance defined by both Mathematics and Thai Language tests are presented here. Overall, approximately 37 % of the students (n = 168) had poor school performance in test scores of both Mathematics and Thai Language. A history of malaria infection, in terms of malaria infection, number of malaria attacks, age at first malaria infection, and duration since the last attack, was not significantly associated with school performance, even after adjusting for potential confounders (Table 5).

**Table 5:** Associations between other factors with school performance in test scores of mathematics/Thai Language

Variable	School performance in test score of mathematics/Thai Language		Crude OR	Adjusted OR
	Poor (n = 168)	Not poor (n = 289)		
History of malaria infection				
Non-malaria infected	117 (36.3)	205 (63.7)	1.00	1.00
Malaria infected	51 (37.8)	84 (62.2)	1.06 (0.70–1.61)	0.90 (0.56–1.45)
Number of malaria attacks				
0	117 (36.3)	205 (63.7)	1.00	
1	25 (26.8)	43 (63.2)	1.02 (0.59–1.75)	
>1	168 (26.8)	41 (61.2)	1.11 (0.65–1.91)	
Age at first malaria infection				
>4 years	26 (35.1)	48 (64.9)	1.00	
0–4 years	25 (41.0)	36 (59.0)	1.28 (0.64–2.58)	
Duration since last malaria attack				
Non-malaria infected	117 (36.3)	205 (63.7)	1.00	
≤2 years	12 (52.2)	11 (47.8)	1.91 (0.81–4.47)	
3–4 years	8 (24.2)	25 (75.8)	0.56 (0.24–1.28)	
≥5 years	31 (39.2)	48 (60.8)	1.13 (0.68–1.87)	
Sex				
Female	48 (21.8)	172 (78.2)	1.00	1.00
Male	120 (50.6)	117 (49.4)	3.67 (2.44–5.53)	3.38 (2.22–5.17)
Ethnicity				
Thai	28 (33.7)	55 (66.3)	1.00	
Karen	140 (37.4)	234 (62.6)	1.17 (0.71–1.94)	
Age (years)				
12–17 years	67 (31.5)	146 (68.5)	1.00	1.00
6–11 years	101 (41.4)	143 (58.6)	1.54 (1.04–2.26)	2.04 (1.31–3.18)
Father’s education				
Educated	62 (33.5)	123 (66.5)	1.00	
Uneducated	106 (39.0)	166 (61.0)	1.27 (0.86–1.87)	
Mother’s education				
Educated	42 (31.3)	92 (68.7)	1.00	
Uneducated	126 (39.0)	197 (61.0)	1.40 (0.91–2.15)	
Family income per year (baht)				
≥30,000	47 (31.1)	104 (68.9)	1.00	
<30,000	121 (39.5)	185 (60.5)	1.45 (0.96–2.19)	
Birth weight				
<2500 g	44 (35.8)	79 (64.2)	1.00	
≥2500 g	117 (37.7)	193 (62.3)	1.09 (0.70–1.68)	
Gestational age				
≥36 weeks	115 (36.5)	200 (63.5)	1.00	
<36 weeks	47 (39.2)	73 (60.8)	1.12 (0.73–1.72)	
Parasite infection				
Infected	36 (36.7)	62 (63.3)	1.00	
None	39 (37.5)	65 (62.5)	1.03 (0.58–1.83)	
School absenteeism				
<1 week	129 (34.7)	243 (65.3)	1.00	1.00
≥1 week	39 (45.9)	46 (54.1)	1.60 (0.99–2.57)	1.72 (1.01–2.92)
Emotional intelligence				
Normal	83 (30.0)	194 (70.0)	1.00	1.00
Low	85 (47.2)	95 (52.8)	2.09 (1.42–3.09)	2.21 (1.43–3.41)
Nutritional status				
Weight for age				
Non poor nutritional status	146 (36.0)	260 (64.0)	1.00	
Poor nutritional status	22 (43.1)	29 (59.9)	1.35 (0.74–2.44)	
Height For Age				
Non poor nutritional status	141 (36.0)	251 (64.0)	1.00	
Poor nutritional status	27 (41.5)	38 (58.5)	1.26 (0.74–2.16)	

Male students were more than three times more likely than female students to have poor school performance (adjusted OR 3.38, 95 % CI 2.22–5.17). Poor school performance was also observed more among younger students (aged 6–11 years) than in those 12–17 years old. In addition, students who had more school absenteeism (≥1 week) were about 1.7 times more likely to have poor school performance. Low emotional intelligence was also associated with poor school performance (adjusted OR 2.21, 95 % CI 1.43–3.41). However, ethnicity of students, parents’ education, family income, perinatal history, history of parasitic infection, and nutritional status were not significantly associated with poor school performance (Table 5).

## Discussion

Children are considered as a high-risk group for malaria infection. There are concerns regarding the consequences of malaria infection in small children, since it may affect the neurodevelopment of infants. Previous studies have shown that malaria infection may affect cognitive function and lead to impairments to memory and language function [10–14]. These studies were mostly conducted in areas of high malaria transmission, where severe malaria among children is more common, or evaluated cognitive function after malaria infection over the short term (1–2 years). In the present study, the long-term effect of malaria infection on cognitive function, as indicated by school performance in mathematics and language, was assessed among children living in an area of low malaria transmission.

Although malaria transmission is considered low in this area, 30 % of school children had experienced at least one malaria infection and about half had had a malaria infection during the first four years of life, the critical period for brain development [26, 27]. However, the results of the study showed that childhood malaria infection was not associated with poor school performance. In addition, duration since the last malaria infection was also not significantly associated with school performance, although those who had last malaria attack within two years were likely to have poor school performance (OR 1.91). Since it was longer than three years since the last malaria infection for the majority (80 %) of malaria-infected children in this study, these findings may reflect the true long-term consequences of malaria infection. These results are inconsistent with those of other studies that demonstrated a short-term impact of malaria infection on school performance [14–18]. Although some previous studies have also reported a significant long-term impact of malaria on school performance [8, 9], these studies were conducted among children with severe malaria, whereas all malaria cases observed in this study were uncomplicated infection. This suggests that the long-term effects on cognitive function might depend on the severity of malaria infection.

Numerous school children in this study had undergone multiple malaria episodes since birth. The data showed that the mean test scores in both mathematics and language were significantly associated with the number of malaria attacks. These findings are consistent with those reported from a study conducted in Sri Lanka [14]. However, the effect of multiple malaria attacks was not observed when poor school performance was classified using a standardized score.

This study showed that males had a greater tendency than females to perform poorly in school. In general, girls are reported to have greater concentration levels during study than boys, perhaps because boys usually are more inclined towards playing or outdoor activities than learning in the classroom. Therefore, girls may perform better than boys in class examinations [28, 29]. The age of students was also associated with school performance in this study. Young children may demonstrate less maturity and lower concentration when learning in comparison with older children.

School performance was also associated with absenteeism. As expected, students who were absent from school for a total of one week or more over a semester had a higher possibility of poor school performance than those who were absent for less than one week. This finding is consistent with that of a previous study conducted in a malaria-endemic area in the Brazilian Amazon [17]. School absenteeism may lead to loss of knowledge provided in the classroom, leading to absent students academically lagging behind other students in the same class.

Low emotional intelligence was associated with poor school performance. The emotional intelligence of children may be affected by many cultural, social, and environmental factors. Emotional intelligence may influence the concentration, attention, and behaviour of children during school, and affect school performance [30–34].

Other potential predictive factors (confounders) for poor school performance were also explored in this study, including socioeconomic status, parents’ education, perinatal history, history of intestinal parasitic infection, and current nutritional status. These findings suggested that these factors were not associated with the school performance of students. In this geographic area the population is fairly homogeneous in terms of socioeconomic status, culture, and lifestyle; these factors may therefore not make a difference in the performance of children in school. Although the school participating in this study is the only one in this rural area, it has very good school health programs. Intestinal parasitic infection has been periodically detected and treated. To improve their nutritional status, it is routinely assessed yearly for all students; therefore, chronic malnutrition is rare. Almost 80 % of students in this school had normal nutritional status, which is higher than that reported for other schools in rural areas of Thailand [21]. This fact supports the study results that current nutritional status was not associated with school performance in this population.

However, the cause of poor school performance is multifactorial. Although several potential confounders have been explored and adjusted in this study, some variables may be left unadjusted, such as underlying diseases of students and the learning environment outside school. However, most of the students in the study were healthy; chronic diseases were not observed in this study population. In addition, variation in the learning environment outside school in this rural area is less likely than in urban areas where competition and learning opportunities are greater. This study was conducted in a setting of low malaria transmission where the majority of the population is Karen, a minority group. Therefore, results from this study may not be generalizable to children living in hyperendemic areas or in other urban settings.

The test scores and nutritional status data collected in this study were based on school reports. Although the paper examinations were developed following a standard protocol and a same set of paper examination was given to students in the same grade, the test scores might still vary depending on teachers’ assessment. This would affect the reliability of test scores when comparing among different classroom. However, the standardized scores, rather than the raw scores, were used to classified school performance status of students to avoid bias due to variation of grading by different teachers. Errors may also occur when measuring weight and height, especially when performed by different persons or different equipment. However, weight and height data in this school were collected by trained teacher using the same measurement tools; this would ensure the reliability of nutritional status data collected in the study.

In this study, data on history of malaria infection in students were obtained from the only malaria clinic in the area. The village census record that has been regularly updated was used to identify students who permanently live in the area but did not have malaria infection. Using these data sources helped to ensure the validity of the past malaria status of the subjects. In addition, school performance status was classified using a standardized score to avoid bias that might occur because of variation in standard grading by different teachers in different classrooms. Moreover, this study tracked the history of malaria infection since birth, which provides information on the long-term consequences of uncomplicated malaria in children living in an area of low malaria transmission.

## Conclusions

Childhood malaria infection may affect cognitive function and lead to impairment of memory and language functions. However, the association between childhood malaria infection and school performance was not significance in this study, when the majority of malaria infected children previously had uncomplicated malaria infection in the past three years. These findings suggested that, in low malaria endemic area, the educational consequence after uncomplicated malaria may be less and may not be prolonged.
